# The Relative Importance of Shear Forces and Surface Hydrophobicity on Biofilm Formation by Coccoid Cyanobacteria

**DOI:** 10.3390/polym12030653

**Published:** 2020-03-12

**Authors:** Sara I. Faria, Rita Teixeira-Santos, Maria J. Romeu, João Morais, Vitor Vasconcelos, Filipe J. Mergulhão

**Affiliations:** 1 LEPABE—Department of Chemical Engineering, Faculty of Engineering, University of Porto, Rua Dr. Roberto Frias, 4200-465 Porto, Portugal; sisf@fe.up.pt (S.I.F.); ritadtsantos@fe.up.pt (R.T.-S.); mariaromeu@fe.up.pt (M.J.R.); 2 CIIMAR—Interdisciplinar Centre of Marine and Environmental Research, University of Porto, Terminal de Cruzeiros do Porto de Leixões, Avenida General Norton de Matos, S/N, 4450-208 Matosinhos, Portugal; jmorais@ciimar.up.pt (J.M.); vmvascon@fc.up.pt (V.V.); 3 FCUP—Faculty of Sciences, University of Porto, Rua do Campo Alegre, 4069-007 Porto, Portugal

**Keywords:** marine biofouling, biofilm formation, coccoid cyanobacteria, hydrodynamic conditions, surface hydrophobicity, polymer coating

## Abstract

Understanding the conditions affecting cyanobacterial biofilm development is crucial to develop new antibiofouling strategies and decrease the economic and environmental impact of biofilms in marine settings. In this study, we investigated the relative importance of shear forces and surface hydrophobicity on biofilm development by two coccoid cyanobacteria with different biofilm formation capacities. The strong biofilm-forming *Synechocystis salina* was used along with the weaker biofilm-forming *Cyanobium* sp. Biofilms were developed in defined hydrodynamic conditions using glass (a model hydrophilic surface) and a polymeric epoxy coating (a hydrophobic surface) as substrates. Biofilms developed in both surfaces at lower shear conditions contained a higher number of cells and presented higher values for wet weight, thickness, and chlorophyll *a* content. The impact of hydrodynamics on biofilm development was generally stronger than the impact of surface hydrophobicity, but a combined effect of these two parameters strongly affected biofilm formation for the weaker biofilm-producing organism. The antibiofilm performance of the polymeric coating was confirmed at the hydrodynamic conditions prevailing in ports. Shear forces were shown to have a profound impact on biofilm development in marine settings regardless of the fouling capacity of the existing flora and the hydrophobicity of the surface.

## 1. Introduction

Marine biofouling is an area of intense research particularly due to the considerable economic impacts on marine transport. Biofouling on ship hulls increases frictional drag and may result in a fuel consumption increase ranging from 6% to 45%, depending on the size of the vessel [1,2,3,4]. This is associated with increased emissions of greenhouse gases and environmental pollution [3]. In addition to the problems associated with frictional drag, marine biofouling poses other environmental problems such as the introduction of nonindigenous species in different habitats including the transport of pathogenic species [5,6].

Biofouling by macrofouling organisms such as bryozoans, mollusks, polychaeta, tunicates, coelenterates, or fungi occurs after biofilm formation by microfouling organisms such as cyanobacteria and diatoms which are early colonizers [7,8,9,10]. Thus, it has been suggested that reducing biofilm formation may be a good strategy to delay macrofouling [11,12,13].

Port authorities in different countries are moving towards a “clean hull” policy where vessels must provide evidence of biofouling management before they arrive [14,15,16]. The enforcement of these policies is likely to be more intense in large-sized vessels whereas small recreational vessels may be subjected to less stringent control and may have a significant impact on the introduction of nonindigenous species. It has been recognized that around 87% of nonindigenous marine species in New Zealand are associated with biofouling on international vessels [17,18] and small recreational vessels may play an important role in this process as compliance with regulations is harder to guarantee.

Several parameters have been indicated as modulators of biofilm development, including surface hydrophobicity and hydrodynamic conditions [7,19]. Recently, Romeu et al. demonstrated that lower shear forces promoted biofilm formation using different filamentous cyanobacterial strains, while the surface properties had a less pronounced effect [20].

In this work, we have tested a polymeric epoxy resin commonly used to coat the hulls of small recreational vessels (such as powerboats, yachts, and sailing boats) [6,21]. Particularly used in fiberglass hulls, epoxy resins are selected due to their mechanical strength and chemical resistance [22]. Epoxy composites offer improved resistance to fatigue, hull durability, and enable the production of cosmetically attractive surfaces even after exposure to saltwater and ultraviolet light [23]. In addition to the problems in ship hulls, marine biofouling also affects other surfaces. Glass surfaces can be found in underwater windows of boats, flotation spheres, moored buoys, underwater cameras, measuring devices, or sensors [21,24]. Particularly in these latter cases, when the optical properties of glass windows are compromised, these devices produce incorrect readings and require frequent cleaning and maintenance during their operational lifetime [25].

The main goal of this study was to evaluate the relative importance of shear forces and surface hydrophobicity on cyanobacterial biofilm development in marine settings. For that purpose, we have followed biofilm development in defined hydrodynamic conditions (including those that can be found in harbors), using two cyanobacterial coccoid strains with different biofilm-forming capacities and two model surfaces with different hydrophobicity (glass and an epoxy polymeric coating). These surfaces can be found in ship hulls and also in the windows of underwater sensors and measuring devices.

## 2. Materials and Methods 

### 2.1. Surface Preparation

In order to assess the cyanobacterial biofilm development, two different surfaces, glass and a polymeric epoxy resin, were used. Glass coupons (1 × 1 cm; Vidraria Lousada, Lda, Lousada, Portugal) were immersed in a 2% (v/v) TEGO 2000^®^ (JohnsonDiversey, Northampton, United Kingdom) solution, an amphoteric disinfectant used for cleaning and disinfecting surfaces [26], for 20 min [27] under agitation (150 rpm). Then, the coupons were washed in sterile distilled water to remove any remaining disinfectant residues, air-dried, and sterilized by autoclaving (121 °C, 15 min) [28]. For the preparation of epoxy-coated glass surfaces, after the washing procedures, glass coupons were gently coated with 150 µL of epoxy resin and dried in two steps: (i) 12 h at room temperature (approximately 25 °C), and (ii) 3 h at 60 °C, according to the instructions from the manufacturer. The polymeric epoxy resin (produced by HB Química company, Matosinhos, Porto, Portugal) is a commercial resin constituted by HB Eposurf 2 resin and HB Eposurf hardener, in a ratio of 10:3. The detailed composition of these reagents is described in the Supplementary Materials (S1—Composition of the polymeric epoxy resin). Coated coupons were immersed in 70% (v/v) ethanol (VWR International S.A.A., Fontenay-sous-Bois, France) for 20 min to sterilize them, according to the indications from the manufacturer. After drying, the initial weight of each coupon was registered.

The water contact angle of both surfaces was determined in three independent measurements performed at 25 ± 2 °C, by the sessile drop method using a contact angle meter (Dataphysics OCA 15 Plus, Filderstadt, Germany), as described in Gomes et al. 2015 [29]. In each experiment, at least 25 determinations for each material were performed.

### 2.2. Cyanobacterial Strains and Growth Conditions

Cyanobacterial strains were obtained from the Blue Biotechnology and Ecotoxicology Culture Collection (LEGE-CCB) deposited at the Interdisciplinary Centre of Marine and Environmental Research (CIIMAR), Porto, Portugal [30]. *Synechocystis salina* LEGE 00041 (order Synechococcales) was originally obtained from a seawater sample, collected on June 2000, at Espinho beach (41.00847 N 8.646958 W) located in the north coast of Portugal [30]. *Cyanobium* sp. LEGE 06097 (order Synechococcales) was isolated from the intertidal zone, on green macroalga, collected on July 2006, at Martinhal beach (37.01869 N 8.926714 W) located in Vila do Bispo, Portugal [30]. Cyanobacterial cells were grown in 750 mL Z8 medium [31] supplemented with 25 g·L^−1^ of synthetic sea salts (Tropic Marin) and B12 vitamin (Sigma Aldrich, Merck, Saint Louis, MO, USA). Cultures were grown under 14 h light (10–30 mol photons m^−2^ s ^−1^, λ = 380–700 nm)/10 h dark cycles at 25 °C. 

### 2.3. Biofilm Formation

Biofilm assays were performed on 12-well plates (VWR International, Carnaxide, Portugal) under previously optimized conditions [20]. Briefly, transparent double-sided adhesive tape was used to fix the coupons to the wells. The plates were subjected to UV sterilization for 30 min and, then, the sterile coupons were fixed. Each well was incubated with 3 mL of cyanobacterial suspension at a concentration of 1 × 10^8^ cell/mL. Microtiter plates were incubated at 25 °C in an orbital shaker with a 25 mm orbital radius (Agitorb 200ICP, Norconcessus, Ermesinde, Portugal) at 40 and 185 rpm and under alternate light cycles of 14 h light (10–30 mol photons m^−2^ s^−1^)/10 h dark. The selection of the hydrodynamic conditions was based on a previous study describing that a shaking frequency of 185 rpm in this incubator corresponds to an average shear rate of 40 s^−1^ and a maximum of 120 s^−1^, while 40 rpm corresponds to an average shear rate of 4 s^−1^ and a maximum of 11 s^−1^ as determined by computational fluid dynamics [20]. As the shear rate of 50 s^−1^ was estimated for a ship in a harbor [32], and lower shear rates promote marine biofouling [33,34], both hydrodynamic conditions were evaluated. 

Biofilm formation was followed for six weeks (42 days), every seven days. During the incubation period, the culture medium was replaced twice a week. Biofilm formation experiments were performed with two technical replicates and in two independent assays (biological replicates).

### 2.4. Biofilm Analysis

At each sampling point, two coupons of each experimental condition were analyzed concerning (i) the number of biofilm cells, (ii) biofilm wet weight, (iii) biofilm thickness, and iv) chlorophyll *a* content. The biofilm structure was analyzed at day 42 by optical coherence tomography (OCT). Before sampling the culture medium was carefully removed and, then, the coupons were gently rinsed with a sterile sodium chloride solution (8.5 g·L^−1^, VWR International, Carnaxide, Portugal) in order to remove loosely attached cyanobacteria. 

#### 2.4.1. Cyanobacterial Cell Counting

Cyanobacterial cells were detached from the coupons by dipping each coupon in 2 mL of 8.5 g·L^−1^ sodium chloride solution and vortexing for 3 min at maximum power. Then, 10 µL of cellular suspension was placed on each side of a Neubauer chamber and observed under the microscope (Nikon Eclipse LV100 microscope, Nikon Corporation, Tokyo, Japan). After vortexing, the coupons were observed by microscopy in order to confirm complete cell detachment.

#### 2.4.2. Biofilm Wet Weight and Thickness

To determine the biofilm wet weight, coupons were detached from the wells with a sterile tweezer and weighted. Biofilm wet weight was obtained by the difference between initial coupon weight, determined prior to inoculation, and the weight after sampling.

Biofilm thickness was assessed using a Nikon Eclipse LV100 microscope coupled to a joystick (Prior Scientific Ltd., Cambridge, UK), connected to a camera (Nikon digital sight DS-RI 1, Tokyo, Japan), and analyzed using the NIS-Elements AR (Advanced Research) 4.13.05 software package. This tool features fully automated acquisition and device control through multi-dimensional image acquisition and analysis. For each coupon, a minimum of five representative independent fields were analyzed to obtain accurate and reproducible results.

#### 2.4.3. Chlorophyll *a* Quantification

Chlorophyll *a* quantification is a common method to estimate the biomass on marine environments because this pigment is unique and predominant in all groups of cyanobacteria [35]. Detached cells were harvested by centrifugation (3202× *g*, for 5 min at room temperature) and the supernatant discarded. Since chlorophyll pigments are light-sensitive, the following chlorophyll extraction procedures were performed in the dark, as previously reported [20]. Briefly, 2 mL of 99.8% methanol (VWR International, Carnaxide, Portugal) was added to the pellet for chlorophyll extraction. Then, cell suspensions were incubated at 4 °C, during a period of 24 h for a maximal chlorophyll *a* extraction. The absorbance at 750 nm (turbidity), 665 nm (chlorophyll *a*) and 652 nm (chlorophyll *b*) were measured on a V-1200 spectrophotometer (VWR International China Co., Ltd., Shanghai, China). The chlorophyll *a* concentration (μg·cm^−2^) was calculated using the following Equation (1) [36].

*Chl a* (μg·mL^−1^) = 16.29 × *A*^665^ − 8.54 × *A*^652^ (1)

#### 2.4.4. Optical Coherence Tomography

On day 42, the biofilms were imaged by OCT using a Thorlabs Ganymede instrument (Thorlabs GmbH, Dachau, Germany) with a central wavelength of 930 nm. After the gentle rinsing, the wells were filled with 3 mL of a sterile sodium chloride solution (8.5 g·L^−1^) and imaged. The captured volume was 3.66 × 1.52 × 2.98 mm^3^ (509 × 313 × 1024 pixels). The refractive index was set to 1.40, since this value produced optimal results in a previous study [20]. For each coupon, 2D imaging was performed with a minimum of five fields of view to ensure the accuracy and reproducibility of the results obtained.

### 2.5. Data Analysis

Descriptive statistics were used to compute mean and standard deviation for sample parameters (the number of biofilm cells, biofilm wet weight, biofilm thickness, and chlorophyll *a* content). Results were presented as the percentage increase between shear forces (obtained at 40 and 185 rpm). 

Data analysis was performed using the GraphPad Prism^®^ for Windows, version 6.01 (GraphPad Software, Inc., San Diego, CA, USA). Since the distribution of some variables was not normal, both parametric and nonparametric tests were used. Student’s t-test was used to compare biofilm formation under lower and higher shear forces, either for glass or epoxy-coated glass surfaces. For the determination of the impact of the hydrodynamic conditions and surface hydrophobicity on biofilm formation, the Mann–Whitney test was used (data shown in the Supplementary Materials). Significant results were considered for *p*-values < 0.05. 

The impact of the hydrodynamic condition and surface hydrophobicity on biofilm development was estimated for each analyzed parameter (the number of biofilm cells, wet weight, thickness, and chlorophyll *a* content) and represented in radar charts. Radar charts were divided into four quadrants, where each one depicts the average values obtained in each sampling point (days) under the following experimental conditions: Q1) glass at 40 rpm (*Gla*/40), Q2) epoxy-coated glass at 40 rpm (*Epx*/40), Q3) epoxy-coated glass at 185 rpm (*Epx*/185), and Q4) glass at 40 rpm (*Gla*/185). The impact of the hydrodynamic conditions was calculated by subtracting the values obtained at different shear forces for both glass (Q1 vs. Q4) and epoxy-coated glass (Q2 vs. Q3); whereas the impact of the surface hydrophobicity was determined by subtracting the values obtained for two different surfaces at lower shear (Q1 vs. Q2) and higher shear (Q4 vs. Q3). All positive differences were considered as increments resulting from hydrodynamic condition or surface hydrophobicity and represented by a colored area (hydrodynamic effect—yellow area; surface effect—blue area). The combined effect (green area) has been plotted whenever the surface effect overlapped the hydrodynamic effect.

## 3. Results

In this study, we investigated the impact of shear forces and surface hydrophobicity on biofilm development by two coccoid cyanobacteria. The different shear forces were obtained by using distinct shaking frequencies (40 and 185 rpm) in an orbital incubator generating average shear rates of 4 and 40 s^−1^, respectively, as determined by computational fluid dynamics [20]. Surface hydrophobicity was evaluated by determining the water contact angle. A value of 39.5° was obtained for glass whereas for the epoxy-coated surface it was 90.2° (shown on Supplementary Material, S2—A representative image of water contact angle measurement). While glass is clearly hydrophilic, the epoxy-coated surface is slightly hydrophobic [37,38].

**Figure 1:**
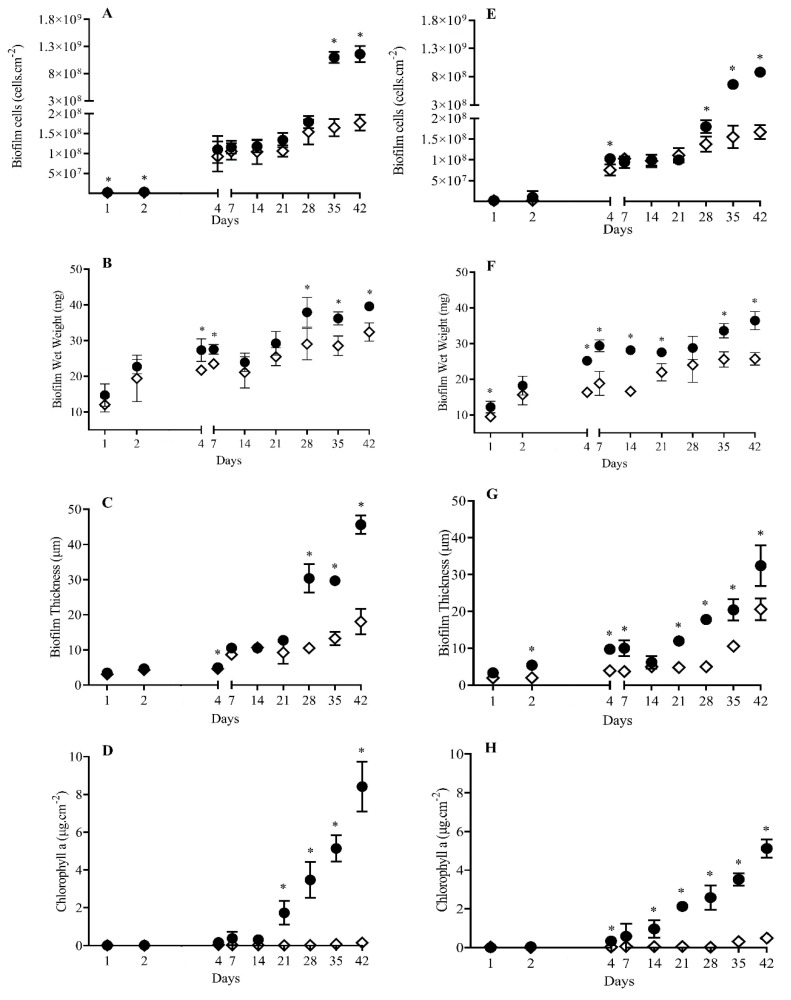
Evaluation of the influence of hydrodynamic conditions on biofilm development of *Synechocystic salina* LEGE 00041 for 42 days, on glass (**A**–**D**) and epoxy-coated glass (**E**–**H**), respectively. The analyzed parameters refer to biofilm cells (**A** and **E**), biofilm wet weight (**B** and **F**), biofilm thickness (**C** and **G**), and chlorophyll *a* (**D** and **H**) at two different hydrodynamic conditions (● 40 rpm; ◊ 185 rpm). Symbol * indicates significant results for *p*-values < 0.05, comparing the two hydrodynamic conditions.

**Figure 2:**
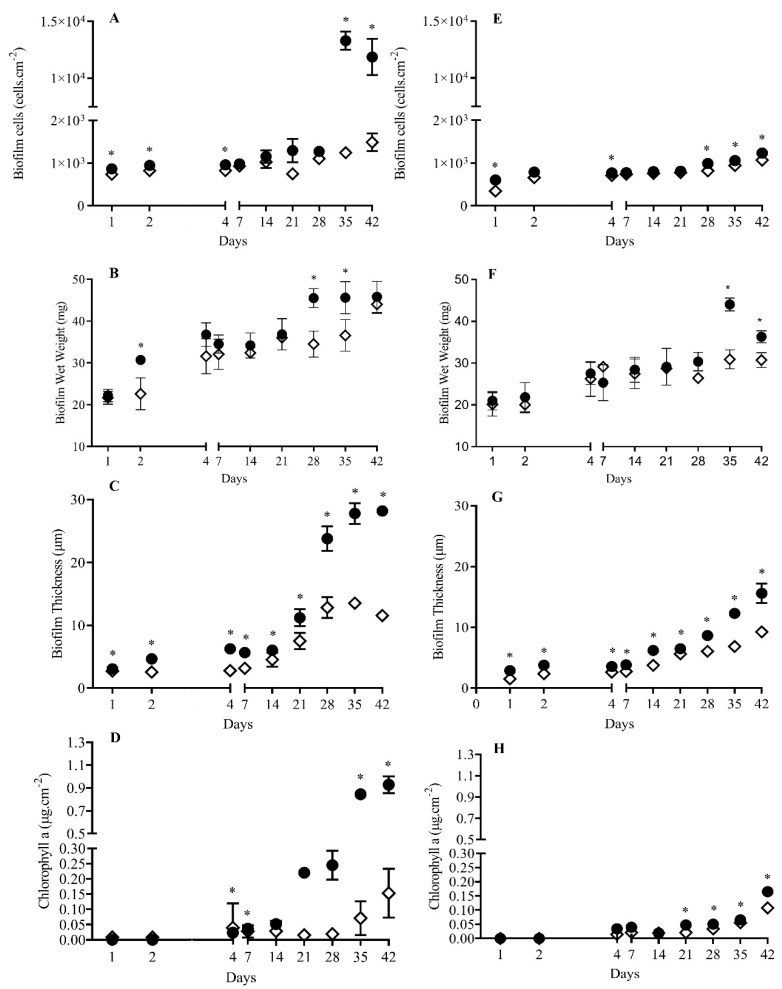
Evaluation of the influence of hydrodynamic conditions on biofilm development of *Cyanobium* sp. LEGE 06097 for 42 days, on glass (**A**–**D**) and epoxy-coated glass (**E**–**H**), respectively. The analyzed parameters refer to biofilm cells (**A** and **E**), biofilm wet weight (**B** and **F**), biofilm thickness (**C** and **G**), and chlorophyll *a* (**D** and **H**) at two different hydrodynamic conditions (● 40 rpm; ◊ 185 rpm). Symbol * indicates significant results for *p*-values < 0.05, comparing the two hydrodynamic conditions.

For both *S. salina* (high biofilm former) and *Cyanobium* sp. (low biofilm former), the number of biofilm cells was higher at lower shear for all the time points tested. Biofilms developed on glass at lower shear displayed on average a higher number of cells of *S. salina* (35%, *p* < 0.05 for 44.4% of the time points, Figure 1A) and *Cyanobium* sp. (32%, *p* < 0.05 for 55.6% of the time points, Figure 2A).

In turn, biofilms formed on the epoxy-coated glass surface at lower shear also had on average a higher number of cells of *S. salina* (31%, *p* < 0.05 for 44.4% for the time points, Figure 1E) and *Cyanobium* sp. (14%, *p* < 0.05 for 55.6% for the time points, Figure 2E).

Biofilms formed on glass at lower shear had, on average, a higher mass for *S. salina* (17%, *p* < 0.05 for 55.6% of the time points, Figure 1B) and *Cyanobium* sp. (12%, *p* < 0.05 for 33.3% of the time points, Figure 2B). On epoxy-coated glass, increased wet weight values were also obtained at low shear for *S. salina* (26%, *p* < 0.05 for 77.8% of the time points, Figure 1F) and *Cyanobium* sp. (10%, *p* < 0.05 for 22.2% of the time points, Figure 2F).

Likewise, biofilms developed on glass at lower shear displayed, on average, a higher thickness for *S. salina* (28%, *p* < 0.05 for 44.4% of the time points, Figure 1C) and *Cyanobium* sp. (41%, *p* < 0.05 for all the time points tested, Figure 2C). On epoxy-coated glass, biofilm thickness was also, on average, higher at lower shear for *S. salina* (52%, *p* < 0.05 for 77.8% of the time points, Figure 1G) and *Cyanobium* sp. (34%, *p* < 0.05 for all the time points tested, Figure 2G).

In addition, biofilms formed on glass at lower shear had, on average, a higher content of chlorophyll *a* for *S. salina* (80%, *p* < 0.05 for 44.4% of the time points, Figure 1D) and *Cyanobium* sp. (73%, *p* < 0.05 for 44.4% of the time points, Figure 2D). Chlorophyll *a* content produced on epoxy-coated glass at lower shear was also, on average, higher for *S. salina* (95%, *p* < 0.05 for 66.7% of time points, Figure 1H) and *Cyanobium* sp. (35%, *p* < 0.05 for 44.4% for time points, Figure 2H). 

**Figure 3:**
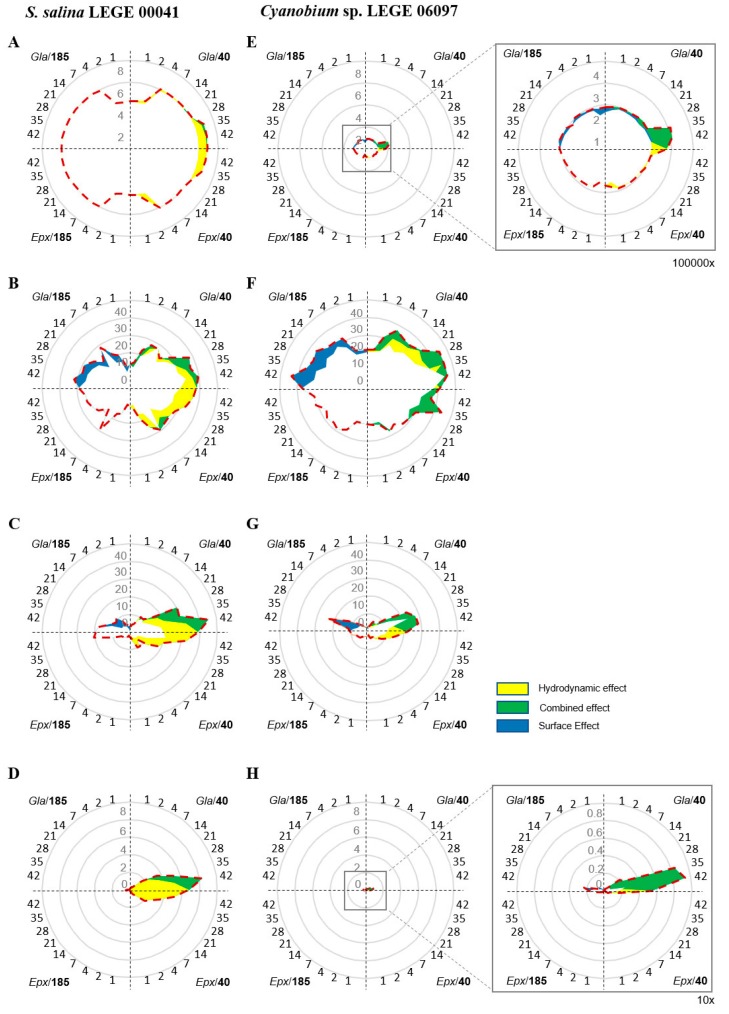
Radar charts representing (**A** and **E**) the number of biofilm cells (Log cells.cm^−2^), (**B** and **F**) biofilm wet weight (mg), (**C** and **G**) biofilm thickness (µm), and (**D** and **H**) chlorophyll *a* content (µg·cm^−2^), for *S. salina* LEGE 00041 and *Cyanobium* sp. LEGE 06097. Average values (previously represented in Figure 1 and Figure 2) are plotted as a dashed line considering the time scale (days) indicated in each quadrant. The following conditions are depicted in each quadrant: Q1: Gla/40 glass at 40 rpm; Q2: Epx/40 epoxy-coated glass at 40 rpm; Q3: Epx/185 epoxy-coated glass at 185 rpm; and Q4: Gla/185 glass at 185 rpm. The hydrodynamic effect calculated by subtracting the values obtained at different shear forces for both glass (Q1 vs. Q4) and epoxy-coated glass (Q2 vs. Q3) is represented by the yellow area. The surface effect determined by subtracting the values obtained for two different surfaces at lower shear (Q1 vs. Q2) and higher shear (Q4 vs. Q3) is represented by the blue area. When these effects overlap, they are represented by the green area. Only positive differences are represented.

For both *S. salina* and *Cyanobium* sp., biofilms formed on glass displayed a slightly higher number of cells compared to those formed on epoxy-coated glass surfaces. Likewise, increased wet weight, thickness, and chlorophyll *a* content values were observed for biofilms developed on glass (Figure 3).

For *S. salina* and for both surfaces, the hydrodynamic conditions had a high impact on the increase of the number of biofilm cells, biofilm wet weight and thickness, and chlorophyll *a* content (Figure 3A–D), as represented by the yellow area. This increase was observed in all the stages of biofilm formation (from day 1 to 42). 

The increase in biofilm wet weight, thickness, and chlorophyll *a* content also resulted from a combined effect between hydrodynamics and surface hydrophobicity (represented by the green area) (Figure 3B–D). However, the pure effect of hydrodynamics was stronger than the combined effect between surface and hydrodynamics (yellow versus green area). Conversely, surface hydrophobicity only had an influence on the wet weight and thickness of biofilms developed at higher shear (Figure 3B–C, blue area).

For *Cyanobium* sp. a combined effect resulting from hydrodynamics and surface hydrophobicity was responsible for a higher number of biofilm cells, biofilm wet weight and thickness, and chlorophyll *a* content (Figure 3E–H, green area). This increment was observed for all sampling points. However, Figure 3E–H show that hydrodynamics had a smaller effect on the increment of these parameters for both glass and epoxy-coated glass surfaces (Figure 3E–H, yellow area), while surface hydrophobicity only induced an increase on these paraments at lower shear (Figure 3E–H, blue area).

**Figure 4:**
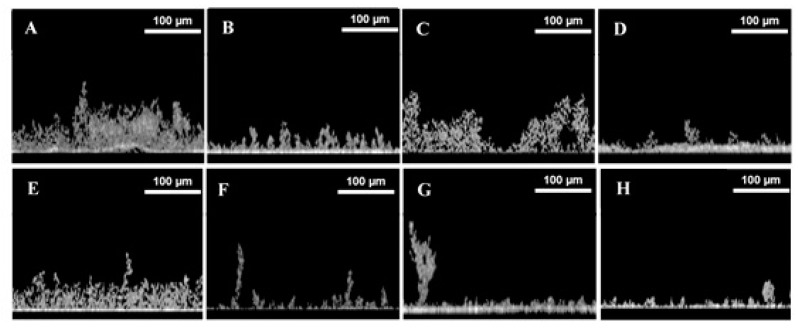
Representative images obtained by optical coherence tomography (OCT) for *S. salina* LEGE 00041 biofilm (**A–D**) and *Cyanobium* sp. LEGE 06097 biofilm (**E–H**), on day 42, on glass at 40 (**A** and **E**) and 185 rpm (**B** and **F**), and on epoxy-coated glass at 40 rpm (**C** and **G**) and 185 rpm (**D** and **H**).

Biofilm structures were evaluated on day 42 using optical coherence tomography for *S. salina* and *Cyanobium* sp. (Figure 4). For both strains, biofilms developed on glass at 40 rpm were more prominent (Figure 4A,E). Moreover, the presence of three-dimensional structures was more noticeable for biofilms formed at lower shear stress for both glass and epoxy-coated glass surfaces (Figure 4A,C,E,G).

## 4. Discussion

Our study clearly demonstrated that shear forces and surface properties have a significant impact on biofilm formation by coccoid cyanobacteria, as confirmed by the number of biofilm cells, biofilm wet weight and thickness, and chlorophyll *a* content.

Cyanobacterial biofilms developed at lower shear (obtained at 40 rpm) presented a higher number of biofilm cells when compared to those developed at higher shear (185 rpm), both on glass and epoxy-coated glass surfaces. Similar results were obtained for wet weight and thickness. This later parameter has a strong impact on the performance of underwater devices [25], and therefore, its assessment during biofilm formation is important not only for the development and maintenance of marine devices, but also to better understand the marine biofilm behaviour. 

Likewise, several studies have proposed the determination of chlorophyll *a* content as a good indicator of cyanobacterial biofilm growth [39,40]. Our results also demonstrated that cyanobacterial biofilms growing at lower shear produced higher amounts of chlorophyll *a*, which is consistent with the higher number of biofilm cells obtained in this condition. These results are corroborated by a previous study demonstrating that biofilm development by filamentous cyanobacteria is also promoted at low shear forces [20].

Concerning the surface hydrophobicity, results demonstrated that biofilms formed on glass displayed a higher number of cells than on epoxy-coated glass. Similarly, the biofilm wet weight and thickness, and chlorophyll *a* content were higher on glass than on epoxy-coated glass. This result suggests that, in this assay, the hydrophilic surface promoted biofilm formation. Other studies referred that adhesion and consequent biofilm formation may occur to a greater extent on hydrophobic surfaces rather than on hydrophilic surfaces [41]. However, according to Mazumder et al., biofilm formation may induce alterations in the hydrophobicity of the substratum surfaces, indicating that bacterial cells already attached can modify the surface properties [42]. 

The tendencies verified for the analyzed parameters were validated for both *S. salina* and *Cyanobium* sp. independently of their capacity to form a biofilm. However, our data analysis demonstrated that, for *S. salina*, the increase in biofilm parameters is mainly due to shear forces (yellow shadowed area is greater than blue shadowed area, Figure 3A–D), whereas for *Cyanobium* sp., a combined effect resulting from the shear force and surface hydrophobicity is responsible for the biofilm development behaviour (green shadowed area prevails over yellow and blue shadowed areas, Figure 3E–H). Therefore, it was shown that shear forces exert a crucial impact on the development of cyanobacterial biofilms, which is important not only in the early stage of biofilm formation but also during maturation. It is known that lower shear forces promote uniform biofilm formation during all stages of its development, while higher shear forces not only promote uneven biofilm formation but may also induce biofilm detachment and deformation [43]. Moreover, it has been reported that higher shear forces cause several functional and morphological changes in biofilms, including quorum-sensing impairment [44,45] and metabolic switching [46], which may hinder their development. 

For strains with low biofilm formation capability, although hydrodynamics also play an important role in biofilm development, surface hydrophobicity becomes more important than in high biofilm producers. In this case, a combined effect between hydrodynamics and hydrophobicity becomes relevant. Surface properties such as hydrophobicity may promote cell/surface interactions that, even in a low magnitude, may facilitate bacterial retention and contribute to biofilm development when combined with lower shear [43]. Furthermore, at a higher shear, these cell/surface interactions seem to become crucial for biofilm formation. 

OCT analysis highlighted the impact of shear forces on cyanobacterial biofilm development, demonstrating that higher biofilm amounts were obtained at lower shear. It was also possible to observe the presence of three-dimensional structures of streamers in biofilms formed under these conditions on both surfaces. According to Drescher et al., the presence of these structures may contribute to biofilm growth by facilitating the capture of new cells and other components to the biofilm [47]. 

In ship hulls, epoxy composites are used due to their strong adhesion to the construction material, high strength, and great chemical resistance [22]. Our results show that the polymeric epoxy resin also has a very good antifouling performance for the specific application that it was designed for. It is known that fouling in ship hulls mainly occurs when the ship is docked due to the lower shear stress when compared to sailing conditions. In this study, we have mimicked the shear forces acting on a ship hull while staying in a port and the results obtained with the polymeric coating suggest that it can decrease biofilm formation. Therefore, it may have the potential to delay hull fouling thus reducing problems associated with frictional drag, fuel consumption, and the introduction of nonindigenous species in different habitats.

## Supplementary Materials

The following are available online at https://www.mdpi.com/2073-4360/12/3/653/s1, Figure S1: A representative image of water contact angle measurement, Table S1: *P*-values obtained for the differences between the hydrodynamic conditions (40 vs. 185 rpm) on biofilm formation (*p*-values < 0.05 are shown in bold), Table S2: *P*-values obtained for the differences between surface hydrophobicity (glass vs. epoxy-coated glass) on biofilm formation (*p*-values < 0.05 are shown in bold).
