# Supplementary material for: The Relative Importance of Shear Forces and Surface Hydrophobicity on Biofilm Formation by Coccoid Cyanobacteria
Source: Polymers (Basel). 2020 Mar 12;12(3):653. doi: 10.3390/polym12030653 (PMC7183090; doi:10.3390/polym12030653)
Supplement: Supplementary file 1 [file polymers-12-00653-s001.pdf]

Supplementary Material

S1 - Composition of the polymeric epoxy resin

The HB Eposurf 2 resin is composed by bisphenol-A- (epichlorohydrin) and epoxy resins (average molecular weight below 700 KDa), Poly (Bisphenol A-co-epichlorohydrin), 4-4`-Isopropylidenediphenol, oligomeric reaction products with 1-cloro-2,3-epoxypropene, phenol, polymer with formaldehyde, oxiranylmethyl ether Poly[(phenyl glycidyl ether)-co-formaldehyde] and 1,6-Hexamethylenediol diglycidyl ether. The HB Eposurf hardener is composed by 3-aminomethyl-3,5,5-trimethylcyclohexylamine, 5-Amino-1,3,3-trimethylcyclohexanemethylamine, mixture of cis and trans, 5-Amino-1,3,3-trimethylcyclohexanemethylamine, mixture of cis and trans, 3-aminimethyl-3,5,5-trimethylcyclohexylamine.

Figure S1 – A representative image of water contact angle measurement. Pictures of water droplets on glass (left) and epoxy-coated glass (right) surfaces.

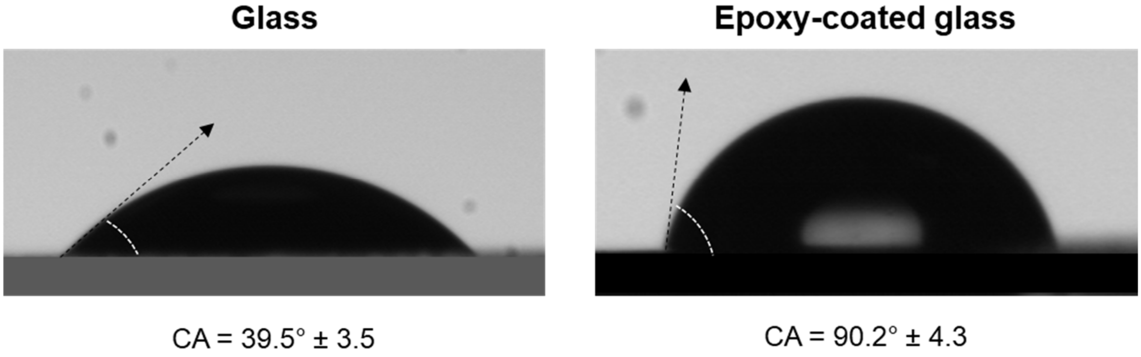

Table S1 – *p*-values obtained for the differences between the hydrodynamic conditions (40 vs 185 rpm) on biofilm formation (*p*-values < 0.05 are shown in bold)

	S. salina 00041		*Cyanobium* sp. 06097	
	**Glass**	**Epoxy-coated glass**	**Glass**	**Epoxy-coated glass**
**Biofilm cells**	0.091	0.275	0.282	**0.048**
**Biofilm wet weight**	0.083	0.685	0.698	0.933
**Biofilm thickness**	**0.018**	**< 0.001**	**0.001**	**0.035**
**Chlorophyll *a* content**	**< 0.001**	**< 0.001**	0.303	0.751

Table S2 – *p*-values obtained for the differences between surface hydrophobicity (glass vs epoxy-coated glass) on biofilm formation (*p*-values <0.05 are shown in bold)

	S. salina 00041		*Cyanobium* sp. 06097	
	**Lower shear**	**Higher shear**	**Lower shear**	**Higher shear**
**Biofilm cells**	0.161	0.589	**0.008**	0.137
**Biofilm wet weight**	0.632	**0.018**	**0.003**	**0.007**
**Biofilm thickness**	0.053	**< 0.001**	**0.001**	0.202
**Chlorophyll *a* content**	0.726	0.208	0.079	0.160
